# 4577 Resistant hypertension potentiates the risk of End-Stage Kidney Disease among African-Americans independent of APOL1 genotype in the Million Veteran Program

**DOI:** 10.1017/cts.2020.144

**Published:** 2020-07-29

**Authors:** Elvis Akwo, Cassiane Robinson-Cohen, Cecilia P. Chung, Peter W.F. Wilson, Christopher O’Donnell, Todd L. Edwards, Csaba P. Kovesdy, Adriana Hung

**Affiliations:** 1 Vanderbilt University Medical Center; 2 Emory University; 3 VA Boston Health Care System; 4 University of Tennessee Health Science Center

## Abstract

OBJECTIVES/GOALS: African-Americans have a 3-fold higher risk of end-stage kidney disease (ESKD) compared to Whites due in part to APOL1 risk alleles. Whether resistant hypertension (RH) magnifies the risk of ESKD among African Americans beyond APOL1 is not known. We examined the interaction between RH and race on ESKD risk and the independent effect of RH beyond *APOL1*. METHODS/STUDY POPULATION: We designed a retrospective cohort of 240,038 veterans with HTN, enrolled in the Million Veteran Program with an estimated glomerular filtration rate (eGFR) >30 ml/min/1.73m^2^. The primary exposure was incident RH (time-varying). The primary outcome was incident ESKD during a 13.5 year follow up: 2004-2017. Secondary outcomes were myocardial infarction (MI), stroke, and death. Incident RH was defined as failure to achieve outpatient blood pressure (BP) <140/90 mmHg with 3 antihypertensive drugs, including a thiazide, or use of 4 or more drugs. Poisson models were used to estimate incidence rates and test additive interaction with race and *APOL1* genotype. Multivariable Cox models (with Fine-Gray competing-risks models as sensitivity analyses) were used to examine independent effects. RESULTS/ANTICIPATED RESULTS: The cohort comprised 235,046 veterans; median age was 60 years; 21% were African-American and 6% were women, with 23,010 incident RH cases observed over a median follow-up time of 10.2 years [interquartile range, 5.6-12.6]. Patients with RH had higher incidence rates [per 1000 person-years] of ESKD (4.5 vs. 1.3), myocardial infarction (6.5 vs. 3.0), stroke (16.4 vs. 7.6) and death (12.0 vs. 6.9) than non-resistant hypertension (NRH). African-Americans with RH had a 2.6-fold higher risk of ESKD compared to African-Americans with NRH; 3-fold the risk of Whites with RH, and 9.6-fold the risk of Whites with NRH [p-interaction<.001]. Among African-Americans, RH was associated with a 2.2-fold (95%CI, 1.86-2.58) higher risk of incident ESKD in models adjusted for *APOL1* genotype and in the subset of African-Americans with no *APOL1* risk alleles, RH was associated with an adjusted 2.75-fold (95% CI: 2.00-3.50) higher risk of incident ESKD. DISCUSSION/SIGNIFICANCE OF IMPACT: RH was independently associated with a higher risk of ESKD and cardiovascular outcomes, especially among African-Americans. This elevated risk is independent of *APOL1* genotype. Interventions that achieve BP targets among patients with RH could curtail the incidence of ESKD and cardiovascular outcomes in this high-risk population. CONFLICT OF INTEREST DESCRIPTION: None.

